# Divalent Metal Ion Transport across Large Biological Ion Channels and Their Effect on Conductance and Selectivity

**DOI:** 10.1155/2012/245786

**Published:** 2012-09-13

**Authors:** Elena García-Giménez, Antonio Alcaraz, Vicente M. Aguilella

**Affiliations:** Laboratory of Molecular Biophysics, Department of Physics, Universitat Jaume I, 12071 Castellón, Spain

## Abstract

Electrophysiological characterization of large protein channels, usually displaying multi-ionic transport and weak ion selectivity, is commonly performed at physiological conditions (moderate gradients of KCl solutions at decimolar concentrations buffered at neutral pH). We extend here the characterization of the OmpF porin, a wide channel of the outer membrane of *E. coli,* by studying the effect of salts of divalent cations on the transport properties of the channel. The regulation of divalent cations concentration is essential in cell metabolism and understanding their effects is of key importance, not only in the channels specifically designed to control their passage but also in other multiionic channels. In particular, in porin channels like OmpF, divalent cations modulate the efficiency of molecules having antimicrobial activity. Taking advantage of the fact that the OmpF channel atomic structure has been resolved both in water and in MgCl_2_ aqueous solutions, we analyze the single channel conductance and the channel selectivity inversion aiming to separate the role of the electrolyte itself, and the counterion accumulation induced by the protein channel charges and other factors (binding, steric effects, etc.) that being of minor importance in salts of monovalent cations become crucial in the case of divalent cations.

## 1. Introduction

The lipid membrane of the cells forms an insulating barrier to the passage of ions, metabolites, and other larger molecules [1]. However, the selective transport of charged solutes and large molecules across the cell membrane is a physiological function necessary for the survival of the cells and hence of the living organisms. That specialized physiological function is carried out by ion channels, a large family of specialized proteins present in all living organisms that open pores of nanometer dimensions in the cell membranes [2–4]. The actual size of the pore determines mostly the specific function of each channel [5]. Thus, narrow channels can efficiently discriminate between different charged species while other processes that require the rapid transport of many ions across the cell membrane are more easily achieved by wider pores also known as mesoscopic channels [6]. This paper focuses on the transport properties of wide channels, in particular on the channel conductance and the ion selectivity. The latter refers here to the ability to favor the passage of certain kind of ions against others when both species are present in solution at the same time (e.g., cations and anions). The fact that the transport through wide channels is passive and multi-ionic makes them suitable for regulating the influx of nutrients and to extrude waste products, necessary in cell metabolism. Their weak selectivity for low-molecular-weight inorganic ions is relevant for several reasons. First, because its comprehension is a first, necessary step to interpret adequately the highly sophisticated mechanisms responsible for the specific selectivity in narrow channels [5]. Second, because the study and understanding of their function have contributed to develop a variety of biotechnological, analytical, and medical applications [7–9].

Mesoscopic channels usually discriminate ions by their charge, that is, the channel is cation selective or anion selective, depending on whether cation or anion transport is favored by the protein [5, 10–12]. The selectivity of these wide channels is mainly regulated by electrostatic interactions between protein ionizable residues and the permeating ions [2]. However, additional factors such as diffusion and even short range interactions may play a role in certain specific cases [6, 13, 14]. Examples of mesoscopic channels extensively studied are bacterial porins like OmpF from *E. coli* [15–17], the voltage-gated anion channel (VDAC) of the outer mitochondrial membrane [18], pore-opening toxins like the alpha-hemolysin channel secreted by *S. Aureus* [19, 20], and antibiotic peptides like alamethicin [21–23]. A common feature of porins is their beta barrel structure. Their hydrophilic environment provides a water-filled pore through which hydrated positive and negative small ions, metabolites like ATP [24] or antibiotic molecules [25], are able to pass. Because of this, porins are also called general diffusion pores [16, 17] and they are weakly selective for small ions. One of them, the bacterial porin OmpF, has been chosen as a model system representative of wide channels in numerous studies. The main reasons are that it is easily genetically modified, overexpressed [26], and crystallized [27]. Besides, its well-known structure obtained at atomic resolution [15] allows establishing a relationship between the channel structure and its function by means of Continuum theories (e.g., Poisson-Nernst-Planck) [28–30] and computational approaches like molecular dynamics (MD) and Brownian dynamics (BD) [28, 31–36]. Furthermore, in this study in particular, where the effects of divalent cations are analyzed in detail, it is especially relevant the fact that the OmpF atomic structure has been reported not only in the presence of salts of monovalent [37] but also in divalent [38] cations.

**Crystallographic Structures of the Ompf Porin**

**Figure 1:**
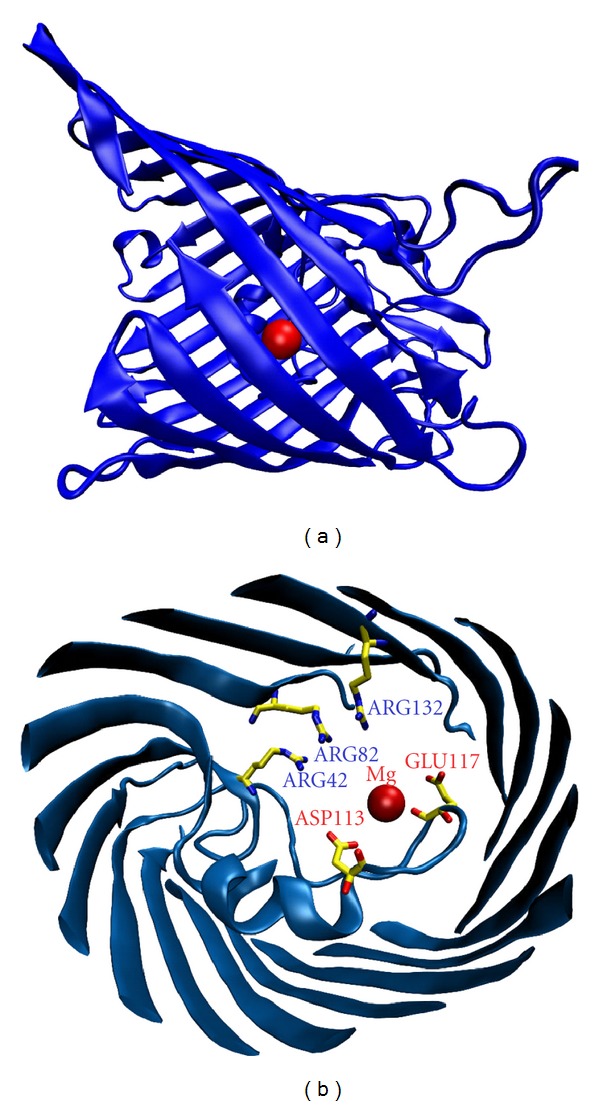
Location of the magnesium ion in the crystal structure of the OmpF channel (PDB code 2ZFG) resolved in 1 M MgCl_2_ solution [38].

OmpF (outer membrane protein F) is a general diffusion porin. It is a homotrimeric protein which forms wide, water-filled, voltage-gated pores in the outer membrane of *E. coli.* Each subunit of the channel has an asymmetric structure, with relatively large entrances of ~4 nm of diameter and a narrow region with diameter ~1 nm at approximately half of the channel length. One of the first crystal structures of the OmpF porin was obtained in 1995 with a resolution of 2.4 Å from X-ray analysis of crystals grown in absence of salt. It is available in the Protein Data Bank (PDB) with 2OMF code [15]. Subsequently, a variety of structures of the OmpF channel and mutants were solved [15, 33, 37–45]. The OmpF structure with PDB code 2ZFG [38] is especially relevant to the present study. It was obtained in 2008 with a resolution of 1.59 Å from crystals grown in a 1 M MgCl_2_ aqueous solution. It displays a Mg^2+^ cation located between the two acid residues of the loop L3 of the structure.  Figure 1 shows the location of the Mg^2+^ cation according to the 2ZFG structure.

The knowledge of the 3D atomic structure of the OmpF porin is a great advantage to establish a relationship between the channel structure and its functional properties. A complete channel characterization requires the combination of different theoretical approaches. Sophisticated methods like MD and BD simulations can provide significant microscopic details such as the crucial effect on the ion transport of the residues present in the narrow constriction of the channel, where a strong electric field transversal to the pore axis is generated separating the pathways for cations and anions along the channel [28, 34]. Using all-atom MD simulations it is also possible to obtain the channel conductance and analyze the behavior of a single protein residue. Alternatively, mean field theories based on the Poison and Nernst-Planck (PNP) equations and the Teorell-Meyer-Sievers model can be used for estimating the conductance and selectivity of the channel under different conditions (salt concentration, solution pH, etc.) and for their comparison with experiments [29, 30, 46, 47].

## 2. Materials and Methods

### 2.1. Channel Reconstitution in Planar Bilayers

A technique widely used for measuring selectivity and conductance in the OmpF channel is its reconstitution in planar lipid membranes. This technique, introduced by Mueller et al. [48] and later improved by Montal and Mueller [49], consists of forming a lipid membrane by the apposition of two lipid monolayers made from a 1% solution of diphytanoyl phosphatidylcholine (DPhPC) (neutral lipid provided by Avanti Polar Lipids, Inc., Alabaster, AL, USA). The lipid bilayer is formed in a micrometric hole (around 80 *μ*m) made on a 15 *μ*m thick Teflon film separating two solutions [50]. The membrane is formed by raising the level of the buffer solution where a small amount of lipid dissolved in an organic solvent has been deposited. Previously, the orifice is pretreated with a 1% solution of hexadecane in pentane to allow adherence. 

**Figure 2:**
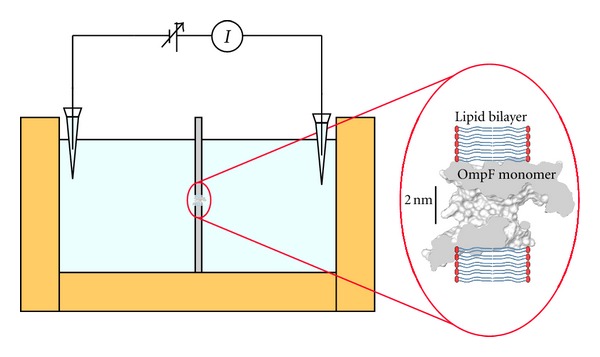
Schematic representation of the experimental setup and reconstitution of the OmpF channel on a lipid bilayer.

The capacitance of the bilayer membrane formed depends on the actual location of the orifices in the film and its size but is always around 80–120 pF. Single-channel insertion is achieved by adding 0.1–0.3 *μ*L of a 1 *μ*g/mL solution of OmpF in a buffer that contains 1 M KCl and 1% (v/v) of Octyl POE (Alexis, Switzerland) to a 2 mL aqueous phase only on the cis side of the membrane. The membrane potential is applied using Ag/AgCl electrodes in 2 M KCl, 1.5% agarose bridges assembled within standard 250 *μ*L pipette tips [50]. Potential is defined as positive when it is greater on the side of protein addition (the *cis* side of the membrane cell).  Figure 2 shows a schematic representation of the experimental setup for the reconstitution of the protein channel on a lipid bilayer.

The membrane chamber and the headstage are isolated from external noise sources with a double metal screen (Amuneal Manufacturing Corp., Philadelphia, PA, USA). To measure the current and apply the potential an Axopatch 200B amplifier (Molecular Devices Corp., Sunnyvale, CA, USA) is used in the voltage-clamp mode. Data are saved directly into the computer memory. Data treatment is done using the PClamp software.

### 2.2. Reversal Potential Measurements and Correction by the Liquid Junction Potential

Channel selectivity is commonly evaluated by measuring the reversal potential (RP). RP is defined as the applied transmembrane voltage that yields zero electric current when there is a concentration gradient across the channel [2]. In the experiments reported here, the RP is obtained as follows. Once a lipid membrane is formed at a given salt concentration gradient, a single OmpF channel is inserted without any applied potential. Next, the channel conductance is checked by applying +50 mV (−50 mV in divalent salts) and later switching the potential polarity. Afterwards, the ionic current through the channel is manually set to zero by adjusting the applied potential. The experimental method used for determining the RP described above introduces two contributions to the electric potential measured (*V*_exp⁡_) [13]: On one hand, the potential difference across each electrode-bridge/solution interface, known as liquid junction potential (LJP); On the other hand, the potential drop across the channel itself, the RP. Hence, the overall LJP has to be subtracted from the raw zero current potential measurement, *V*_exp⁡_,

(1) $$\begin{array}{c} \text{RP}=V_{\exp}-\text{LJP}. \end{array}$$

Usually, salt bridges made of a KCl-concentrated solution are used to measure the channel selectivity following pioneer studies performed under physiological conditions with KCl used as electrolyte [51, 52]. Under those conditions the LJP is small (~1 mV). This is a negligible and usually disregarded quantity as it is comparable to the experimental error of electrophysiology experiments. That is why the protocol is sometimes erroneously extended to altogether different conditions without taking into account that in experiments with other salts (NaCl, LiCl, CaCl_2_, MgCl_2_, etc.) the LJP contribution becomes significant and may be comparable to the actual RP [13]. This repeated oversight, although long noted [53], has led to some inconsistencies in the selectivity data [29, 30, 54–57]. Since direct measurements of LJP are difficult [52, 53], it is necessary to rely on LJP theoretical estimates to determine the actual RP. Assuming ideal electrolyte solutions and linear ion concentration profiles in the junction of the two solutions of salt bridge/and cell compartment, many authors use Henderson's equation [58, 59] to calculate the LJP between two solutions (left (*L*) and right (*R*)):

(2) LJP≡ϕL−ϕR=−(kBTe)∑iziDi[Ci,L−Ci,R]∑izi2Di[Ci,L−Ci,R]ln⁡⁡∑izi2DiCi,L∑izi2DiCi,R,

where *k*_*B*_ and *T* have their usual meaning of the Boltzmann constant and absolute temperature, respectively, and *e* is the elementary charge. *D*_*i*_ denote the ionic diffusion coefficients and *z*_*i*_ and *C*_*i*_ are the ionic valence and concentration, respectively. Under the assumption mentioned above, Henderson's equation is applied for obtaining the LJP in a vast majority of cases of interest and it yields identical results as those obtained using the PNP equations [60]. Specifically, for ion channel measurements, Henderson's equation is a good approximation for estimating LJP contribution to the selectivity measurements [13]. Apart from what has already been said, one has to take into account that when solutions cannot be regarded as ideal or the ionic strength of the two solutions in contact is very different, Henderson's equation becomes a poor approximation, and then LJP calculations demand a proper estimation of ionic activity coefficients and ion mobilities as a function of concentration [61].

### 2.3. Numerical Procedure

We have used the PNP model for computing the conductance and selectivity of the OmpF channel in different salts of monovalent and divalent cations. This model is used here in its one-dimensional version starting from the channel effective fixed charge concentration calculated along the pore. This fixed charge concentration profile is calculated from the 3D electric potential distribution created by the protein-charged residues and it is then averaged along the channel. For calculating this potential it is necessary to know the dissociation constant K_*a*_ (or its equivalent in the logarithmic scale, the pK_*a*_) of each titratable residue inside the protein once the interaction with the protein permanent charges (due to the different electronegativities of the atoms in the molecule) and the other titratable residues are considered. These pK_*a*_ values, known as the apparent pK_*a*_ or effective pK_*a*_ (pK_*a*__eff_), are calculated according the procedure described by Aguilella-Arzo et al. [30] using the UHBD code [62] for two crystallographic structures of the OmpF channel (PDB codes 2OMF and 2ZFG). 

## 3. Effects of Divalent Cations on the Transport Properties of the Channel

### 3.1. Channel Selectivity in Salts of Monovalent and Divalent Cations

The characterization of ion channel selectivity is crucial for understanding the molecular basis of ion transport and establishing a relationship between the structure and the function of the channel [29, 30]. OmpF, like other wide multi-ionic channels, is too large to be specific to a certain ion (as happens in Na or K channels) but still has a clear influence on the permeability. Several experiments aimed to assess ion selectivity [16, 17, 63], as well as MD and BD simulations and continuum electrodiffusion models [28, 34] reveal that the transport of small inorganic ions (K^+^, Na^+^, Cl^−^, etc.) across the channel is principally regulated by the electrostatic interactions between the permeating ions and the channel ionizable residues, in particular (although not solely) by the acidic and basic residues of the channel constriction [28, 31–33]. 

In many cases of interest, RP measurements are used to assess selectivity because the sign of RP provides a quick estimation of the effective charge of the channel: anionic selectivity is associated to a positive net charge and cationic selectivity is directly connected to a negative net charge. Despite being useful as a first estimation, this kind of reasoning must be handled with care [13]. In addition to the electrostatic exclusion/accumulation of ions resulting from their interaction with the protein ionizable residues, another important factor is the difference between the mobilities of the permeating ions themselves within the channel [64]. These diffusional effects as well as other short-range or specific interactions that may take place between the protein residues and mobile ions definitely play a role in the measured RP so it is necessary to design experiments enabling to separate the different contributions as far as possible. The comparison of a number of experiments done with chloride salts of different cations in a variety of conditions and several laboratories allows us to discuss the various sources of ion selectivity in large channels as follows.

- A salt whose cation and anion have the same valence, similar size, and consequently similar bulk mobilities is a suitable electrolyte to study the interaction between ionizable residues and permeating ions because the diffusion effects are negligible. In that respect, KCl is an ideal candidate [29, 30].
- Other salts whose cation and anion present different bulk ion mobilities are appropriate to analyze the contribution of the diffusion potential to the RP measurement. These effects are expected to become more important in salts with ions of different valence.

**Figure 3:**
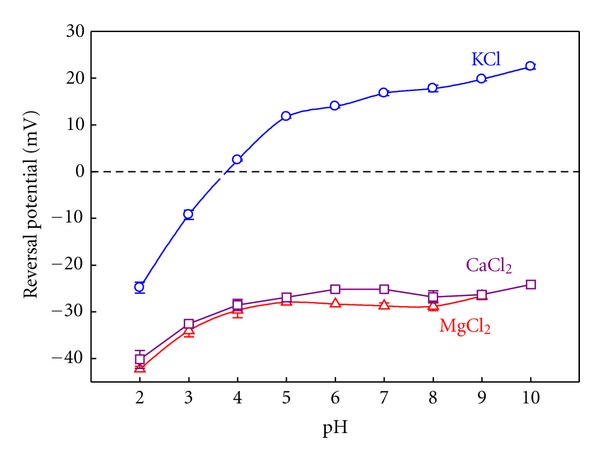
OmpF reversal potential measurements in 0.1/1 M KCl, CaCl_2_, and MgCl_2_ solutions. Adapted with permission from [13].

In view of the OmpF crystal structure obtained from a concentrated solution of MgCl_2_ where a Mg^2+^ ion appears between the two acidic residues (Glu117 and Asp113) of the channel constriction (see Figure 1), experiments done in salts of divalent cations seem appropriate to study possible specific interactions between protein residues and permeating ions that may contribute to the channel RP.  Figure 3 shows RP measurements as a function of the solution pH for KCl, CaCl_2_, and MgCl_2_.

Given that the bulk mobilities of K^+^ and Cl^−^ are very similar, the diffusion potential arising from a 0.1/1 M gradient of KCl can be considered negligible and the RP measurements provide almost direct information about the channel interaction with the permeating ions. Thus, the pH sensitivity seen in the experiments with KCl can be rationalized in terms of the ionization equilibria of all protein ionizable residues (having different pK_*a*_'s) [30]. The overall effect is a different protein net effective charge at each pH as a consequence of the protonation and deprotonation of some protein residues. Therefore, at pH low enough to titrate the acidic residues, the channel is anion selective. That results in a positive net charge of the channel. At pH higher than 3.7 the channel is cation selective, which is consistent with the negative net charge of the channel. This channel pH sensitivity in KCl has previously been analyzed [29, 30, 65, 66] and it is almost independent on the absolute salt concentration.

The RP measurements performed in salts of divalent cations paint a quite different picture [67, 68]. Focusing around neutral pH, the cationic selectivity displayed by the channel in KCl solutions turns into anionic in MgCl_2_ and CaCl_2_. That is why one can think that a charge inversion effect could be taking place on the channel (this occurs when interfacial charges attract counterions in excess of their own nominal charge [68]). In addition, the clear sensitivity to pH shown in KCl disappears both in MgCl_2_ as in CaCl_2_. This change can be explained in terms of a binding process [67]. The presence of divalent cations hinders the protonation of the acidic groups in such a way that an abnormally high amount of protons (and then a lower effective pK_*a*_) is required to neutralize the site. This means that the sensitivity to pH is not lost, but is shifted to lower pH. Interestingly, the pH sensitivity can be restored by lowering the absolute concentration of MgCl_2_ or CaCl_2_ [67]. This result indicates that when the concentration is low enough, the binding of cations is unlikely and has a limited effect on the residue protonation. This is not an exclusive feature of MgCl_2_ and CaCl_2_ salts. The effect of the divalent cations on the sensitivity of the channel to the pH variations and the apparent charge inversion is also observed in a variety of salts of divalent cations such as BaCl_2_ and NiCl_2_ [67].

**Figure 4:**
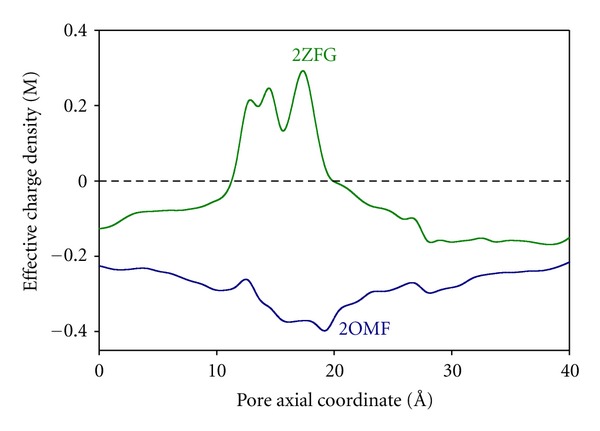
Average fixed charge concentration along the OmpF channel calculated from two OmpF structures: 2OMF (bottom curve) and 2ZFG (top curve). Reprinted with permission from [67].

To get further insight into these RP measurements, we can analyze the connection between charge and selectivity on the basis of the channel 3D structure. The pK_*a*_ calculation leads to a charge concentration profile along the protein for a particular pH [30].  Figure 4 shows the average fixed charge concentration calculated for pH 6 using the two OmpF above mentioned structures: the 3D structure resolved from crystals grown in the absence of salt (2OMF) [15] and the 3D structure obtained from crystals grown in 1 M MgCl_2_ (2ZFG) [38].

The negative charge obtained from the 2OMF structure denoted by the bottom line in Figure 4 is consistent with the cation selectivity of the protein at pH 6 (see Figure 3). The effective charge profile calculated for the 2ZFG structure is very different. The presence of a divalent cation in the narrow part of the channel has a significant effect on the titration of the adjacent residues. As far as this region regulates the selective permeation of ions through the pore, this positive effective charge around the central constriction could be the explanation to the observed anionic selectivity of the channel. However, note that this kind of reasoning is only valid when salts with similar anion and cation mobilities are used and diffusion effects can be negligible. In other salts, the difference between cation and anion diffusivities generates a diffusion potential that necessarily contributes to the measured RP. The diffusion potential would be the electric potential drop across a neutral, ideal pore, devoid of any electrostatic interaction, connecting two solutions at different salt concentrations. Furthermore, in a channel with negative net charge immersed in chloride salts of divalent cations, the diffusion potential and the interfacial Donnan potential may have opposite signs [46]. According to this, the anionic selectivity of the OmpF channel in salts of divalent cations may be simply a consequence of the counterbalance between the diffusional contribution and the channel electrostatic preference for cations.

To get further insight on this selectivity inversion and the role of the diffusion of divalent cations in the channel selectivity, we present a comparison between experiments done in KCl and MgCl_2_ and the 1D PNP model predictions from the effective charge of 2OMF and 2ZFG structures. Note that this original approach based on the structure means an increase in the level of complexity in relation to previous studies using purely phenomenological approaches [13, 14].

**Figure 5:**
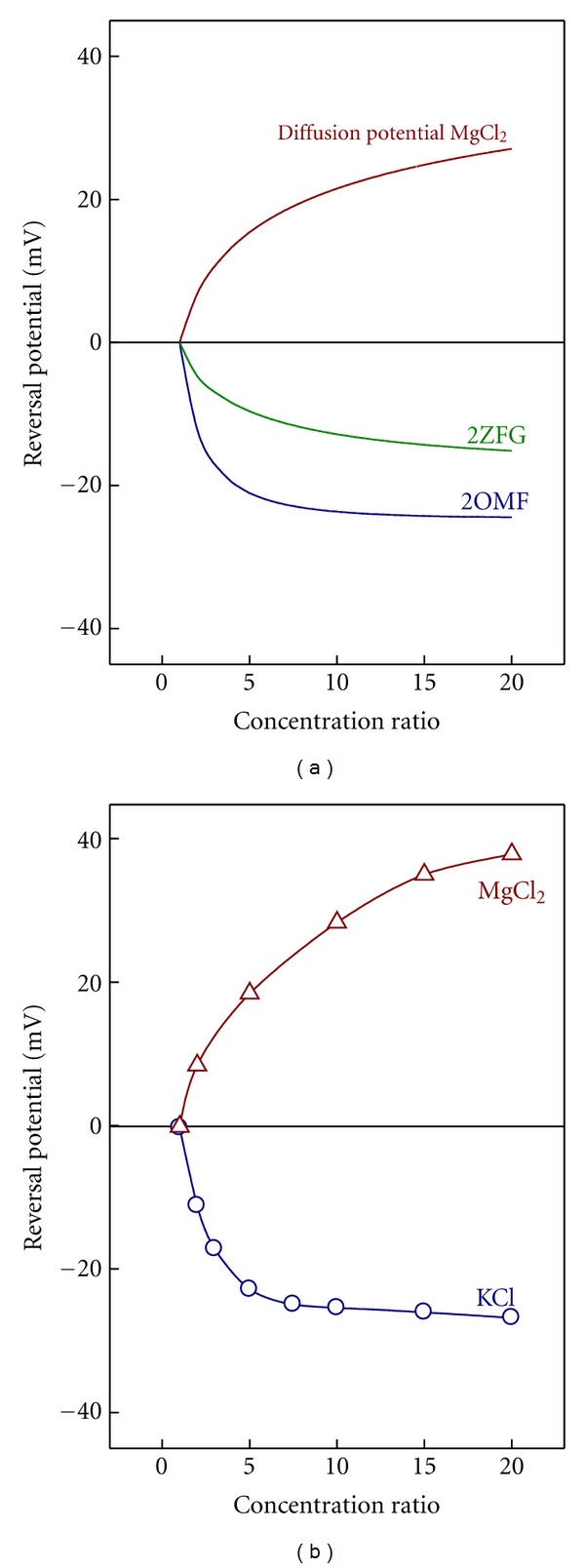
(a) Theoretical predictions of 1D PNP model for the electrostatic exclusion using 2OMF and 2ZFG effective charge profiles from Figure 4 and for the diffusion potential of MgCl_2_. (b) OmpF channel reversal potential measurements in KCl and MgCl_2_ versus concentration ratio (*C*_cis_/*C*_trans⁡_). In all experiments the concentration on trans side is kept constant at 0.1 M and the concentration on cis side is varied. Measurements were done at pH 6.

Figure 5(a) shows the theoretical RP calculated from the 1D PNP model by using the two effective charge profiles displayed in Figure 4 and omitting the difference in the ion mobilities (i.e., considering only the electrostatic exclusion) over a wide range of concentration ratios. The upper plot shows the diffusion potential of MgCl_2_ calculated in a neutral pore using the ion bulk diffusion coefficients. The comparison between Figures 5(a) and 5(b) shows three significant features.

- The model calculations (1D PNP) using the 2OMF effective charge (blue line in Figure 5(a)) correlate very well with the RP measurements in KCl.
- According to the 2ZFG structure the channel net charge is still negative (Figure 4). Thus, the model predictions considering only the electrostatic exclusion effects (green line in Figure 5(a)) do not account for the RP measurements in MgCl_2_, but even give the opposite sign. This indicates that diffusion potentials (brown line in Figure 5(a)) are very significant in the present case and mostly determine the total RP.
- The RP measurements in MgCl_2_ are a little greater than the bulk diffusion potential shown in the figure. This fact has two alternative interpretations, although they do not mutually exclude each other completely.
  1. The negative effective charge of the channel is overcompensated by the divalent cations generating a “charge inversion” phenomenon in the channel [14].
  2. The negative charge of the channel is compensated or almost compensated and the measured RP scales with an effective electric diffusion potential, slightly different from the bulk diffusion potential because of the divalent cations binding effect.

### 3.2. Charge Inversion and Selectivity Inversion

**Figure 6:**
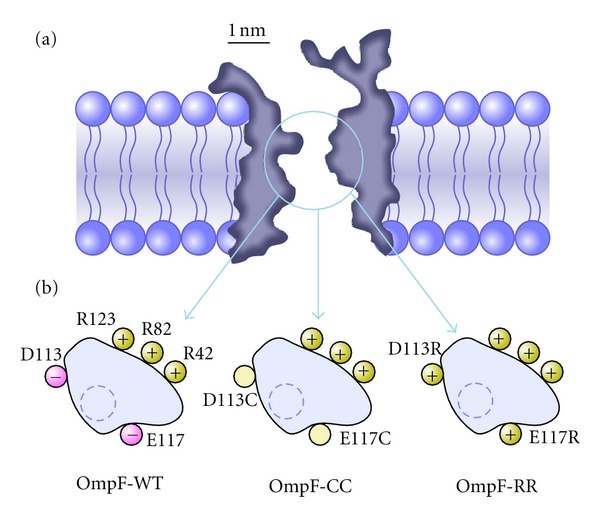
Sketch of the OmpF channel. (a) Longitudinal cross-section. (b) Idealized cross-section of the OmpF channel eyelet for the wild-type (OmpF-WT) protein channel and the mutants with residues Asp113 and Glu117 replaced with cysteines (OmpF-CC) or arginines (OmpF-RR). The dashed contour line represents the hypothetical binding site for a divalent cation based on the atomic structure of OmpF-WT in 1 M MgCl_2_ [38].

The experiments with OmpF channels reported so far make clear that the connection between selectivity and charge is not as obvious as one might think. Thus, the inversion of selectivity found in salts of multivalent cations does not necessarily imply that interfacial charges attract counterions in excess of their own nominal charge, but it can be alternatively caused by a complex interplay of several factors like exclusion, diffusion, and binding. Site-directed mutagenesis has proved to be a powerful tool for understanding the role of certain residues on channel selectivity. For example, it has been reported that the proper mutation of the residues located in the constriction of the OmpF channel can turn it into highly selective to Ca^2+^ ions, with similar transport properties to the Ca^2+^ channel [55, 69]. Having in mind the OmpF crystal structure solved in a 1 M MgCl_2_ where a Mg^2+^ ion appears between the two acidic residues Glu117 and Asp113, we have investigated if the negative charge of those residues is essential to cause an inversion of selectivity. To this end, we compared the reversal potential of the wild-type (OmpF-WT) channel and two mutants in which the above residues had been replaced either by two neutral cysteines (OmpF-CC) or by two positively charged arginines (OmpF-RR). Previous studies using these mutants showed that the dimensions of the narrowest part of the channel are not significantly changed by chemical modification [57], so that additional steric or entropic effects are unlikely.  Figure 6 shows a sketch of the cross-section of the OmpF eyelet in the three cases mentioned. Control experiments of selectivity in monovalent KCl solutions were also carried out.  Table 1 shows the RP measurements in tenfold concentration gradients (1 M cis/0.1 M trans) at pH 6.

Interestingly, the replacement of the two negative residues Asp113 and Glu117 by two neutral ones (see OmpF-CC) does not have a critical effect. The cationic selectivity in salts of KCl is preserved and the selectivity inversion produced by Ca^2+^ ions in OmpF-WT is not removed. Indeed, the anionic selectivity of OmpF-CC is even 50% higher than OmpF-WT. The substitution of Asp113 and Glu117 by two arginines (OmpF-RR) makes the channel anion selective in both salts of monovalent cations (KCl) and divalent cations (CaCl_2_). Therefore, we cannot speak of selectivity inversion in this case. The comparison between OmpF-WT, OmpF-CC, and OmpF-RR suggests that the observed channel preference for anions in salts of divalent cations is not a pure surface effect based on the charge. Rather than that it is probably a joint effect of the long range coulombic interactions between protein and mobile charges and the short-range interaction involving particular functional groups in a precise arrangement [14].

A study using full atomic MD simulations [70] suggested that the inversion of selectivity of OmpF channel can be originated by electrostatic correlations typical of multivalent ions [71]. Thus, the binding of counterions would be an interfacial analogue of the Bjerrum correlations between ions in bulk electrolyte. According to the simulations, the existence of correlations does not require a highly charged interface and depends strongly on the nature (the structure and charge distribution) of the chemical groups present in the interface.

### 3.3. Channel Conductance in Salts of Monovalent and Divalent Cations

**Figure 7:**
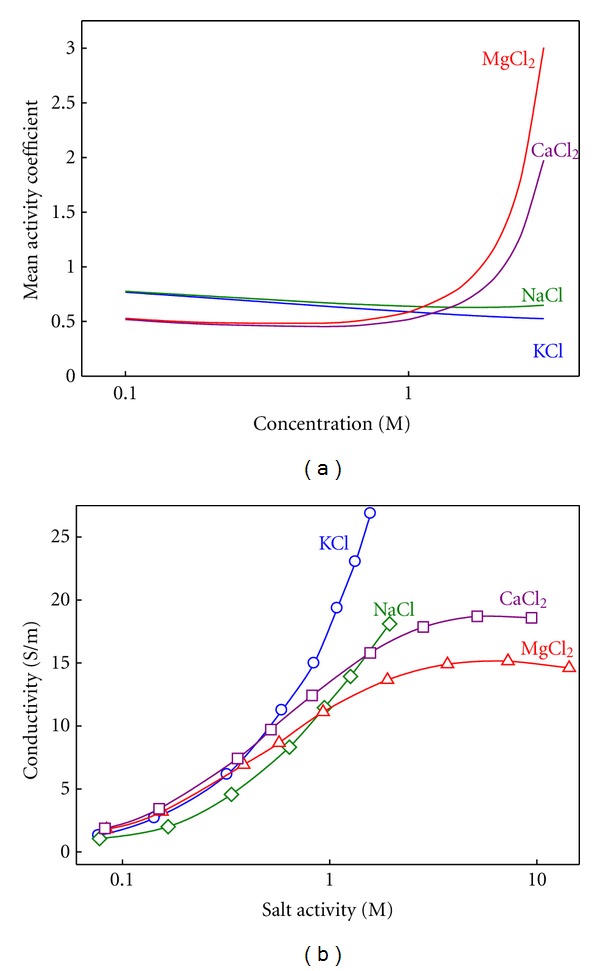
(a) Mean activity coefficient in molar reference as a function of the solution concentration for the electrolytes used in the experiments. (b) Measured conductivity as a function of the electrolyte activity in solution.

The conductance measurements can provide an alternative way of studying the effect of divalent cations on channel transport properties. An initial, necessary step, involves separating channel and electrolyte effects. Otherwise, intrinsic properties of the salt could be incorrectly attributed to the channel action. It is wellknown that in nonideal solutions conductivity increases with the ionic activity rather than with the ion concentration [72]. In addition, the change of the activity coefficient with concentration may be totally different in monovalent cations and divalent cations as is shown in Figure 7(a), where the tabulated values from the literature [73] have been translated from molal to molar scale and later interpolated [73–75].

Figure 7(a) shows marked differences in the activity coefficient between salts of monovalent and divalent cations, especially above 1 M. Whereas in KCl and NaCl it is almost insensitive to concentration, in CaCl_2_ and MgCl_2_ it slightly decreases in the low concentration range, then reaches a minimum and finally shows a steep increase.  Figure 7(b) shows the measured conductivity as a function of the electrolyte activity in solution. In the case of salts of divalent cations, the importance of studying the intrinsic properties of the electrolyte becomes apparent. The solution conductivity of CaCl_2_ and MgCl_2_ saturates with increasing activity. The origin of this saturation is likely to be a strong reduction of the divalent ion diffusion coefficient with the concentration [73]. One might ask about the use of ionic activity here, since ion selectivity has been discussed in terms of ion concentration in the previous section. Note that the study of ion selectivity is done in terms of concentration ratio, not in terms of absolute concentration. Since RP measurements normally involve moderate concentration gradients, the concentration ratio (*C*_cis_/*C*_trans⁡_) [29, 46, 66, 76] is practically equal to the activity ratio (*a*_cis_/*a*_trans⁡_) [72]. Hence, there was no need to invoke the subtle distinction between activity and concentration. However, when the channel conductance is studied as a function of absolute salt concentration, it is necessary to take into account that the activity coefficient may change considerably depending on the salt concentration.

**Figure 8:**
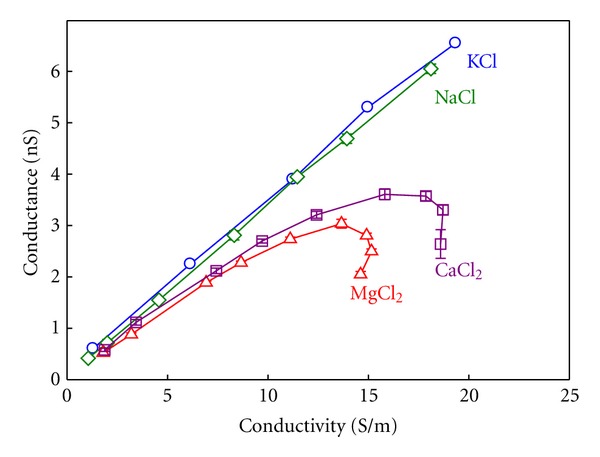
OmpF single channel conductance versus bulk solution conductivity at pH 6. Conductivity is measured in solutions of different salts of concentrations ranging 0.1–3 M. Reprinted with permission from [77].

Channel conductance is obtained from single channel current measurements under an applied potential of +100 mV in symmetrical salt solutions. It is defined as the current per voltage unit (*G* = *I*/*V*). Note that in conductance measurements, there is no correction for the LJP because both electrodes are in contact with solutions with the same concentration. Given that the bulk properties of the electrolytes under study are known (Figure 7(b)), any interaction between the permeating ions and the protein should be seen in the relationship between channel conductance and the respective solution conductivity. In Figure 8 OmpF channel conductance measurements are plotted versus the bulk solution conductivity in a wide range of salt concentration (up to 3 M).

From Figure 8 some conclusions can be drawn:

- A linear correlation is seen between channel conductance and electrolyte bulk conductivity in KCl and NaCl. This would be the expected outcome according to the principle of independent movement of ions applied to the permeation through a channel: the more conductive a solution is the higher channel conductance is measured.
- The conductance-conductivity linear correlation is almost identical in both salts of monovalent cations. This is consistent with a large number of previous studies [13, 14, 30, 35, 54, 66, 76] where it is emphasized that the OmpF channel does not favor the permeation of any particular monovalent ion, which rules out any chemical specificity.
- Two regimes are observed in the relationship between the channel conductance and the solution conductivity in salts of divalent cations: at low and moderate salt concentrations (up to 1 M) conductance scales with bulk conductivity as happens in salts of monovalent cations. This suggests that, in this range, the ion transport is regulated by the electrolyte properties. Above 1 M, the channel conductance as well as the solution conductivity decreases as the concentration increases, breaking the linearity. This indicates that a specific interaction between the divalent cations with the channel can be taking place and shows that current saturation and blocking are not exclusive properties of narrow (single-file) ion channels but may be observed in large, multi-ionic channels like bacterial porins as well.

**Modeling Ion Conductance**

**Figure 9:**
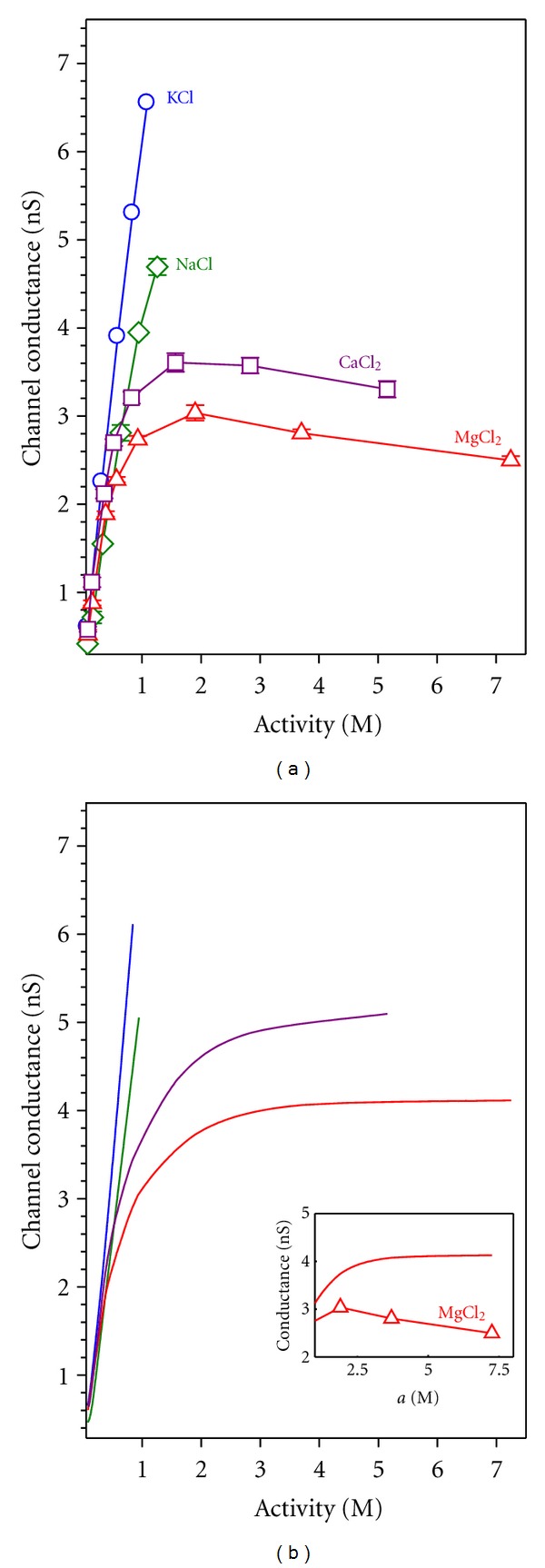
(a) OmpF channel conductance measurements over a wide range of concentrations of several salt solutions. (b) Conductance calculated using the PNP electrodiffusion model and the crystal structure of the OmpF channel as an input. The inset shows a comparison of measured and calculated conductance in highly concentrated MgCl_2_ solutions.

Mean field theories as the 1D PNP model have been used to model the ion transport across the OmpF channel in 1 : 1 salts by using the crystal 3D structure of the porin [30, 47]. In these studies, the tabulated diffusion coefficients for bulk solutions were used and a satisfactory agreement between theory and experiment was found. However, the modeling of channel conductance in 2 : 1 salts cannot ignore the electrolyte intrinsic properties shown in Figure 7. The strong dependence of the electrolyte conductivity on the ion activity can be translated into effective salt-dependent diffusion coefficients as shown in [77].  Figure 9 shows a qualitative comparison between measurements and model calculations of OmpF conductance in different 1 : 1 and 2 : 1 salts.

From the comparison between Figures 9(a) and 9(b) it follows that:

- the measured conductance in KCl and NaCl agree quantitatively, and qualitatively with PNP calculations revealing that at low and moderate concentrations the pore conductance is controlled mainly by the electrolyte properties;
- the saturation in the conductance measurements with the CaCl_2_ and MgCl_2_ concentration is predicted satisfactorily by the PNP model by using the effective salt-dependent diffusion coefficients. This shows that the saturation should be attributed to the electrolyte properties and not to the channel as could be mistakenly thought;
- the decreasing trend of the measured conductance at high enough concentrations in CaCl_2_ and MgCl_2_ (inset of Figure 9(b)) contrasts with the plateau anticipated by the theoretical model. This suggests that a close interaction between the channel residues and the permeating ions not considered in the PNP framework might be involved.

**Conductance and Binding Site of Divalent Cations**

**Figure 10:**
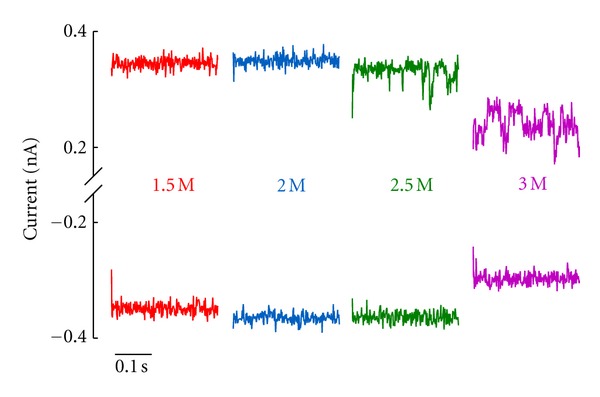
OmpF single-channel current recordings in concentrated solutions of CaCl_2_ for applied voltages of both polarities (+100 mV and −100 mV). Reprinted with permission from [77].

Having in mind the OmpF structure obtained in 1 M MgCl_2_ aqueous solution (2ZFG) [38] showing a Mg^2+^ ion bound to the protein and the experimental evidence of channel selectivity inversion in salts of divalent and trivalent cations [13, 14], one could wonder whether such binding could be behind the conductance decrease observed at high salt concentration of CaCl_2_ or MgCl_2_. Current traces of Figure 10 recorded at several salt concentrations and taken at high sampling frequency can be a clue of whether binding of divalent cations to the protein is modulating the channel conductance. In the lack of further evidence, the traces at high salt concentration point to the existence of substates of lower conductance as one of the causes of the conductance decrease.

Similar observations have been reported for another multi-ionic porin, the lysenin channel, which also displays discrete current changes upon Ca^2+^ addition [78].

## 4. Concluding Remarks

The characterization of biological ion channels in the presence of salts with divalent cations is crucial for understanding the functional relationship of the channel with its environment where several of these metals are present. Multivalent ions are involved in many ion exchange processes for providing an adequate quantity of nutrients to the living cells. In fact, many proteins are specifically expressed in determined conditions for controlling the concentration of certain type of ions in the cell. For example, the MnoP channel (in the outer membrane of *Bradyrhizobium japonicum*) is expressed under manganese limitation for facilitating the translocation of Mn^2+^, but not Co^2+^ or Cu^2+^ [79]. In this short paper, we have analyzed selectivity and conductance measurements in the OmpF porin, considered as representative of other similar large multi-ionic channels, for characterizing the channel function in salts of monovalent and divalent cations. We have shown that salts of divalent cations induce new effects not found in common electrophysiological measurements performed in salts of small monovalent ions. A careful dissection of the different contributions to channel selectivity is needed for a proper characterization of the cationic or anionic preference of the channel because of the specific interactions between divalent cations and protein residues and the significant diffusion potential often involved. Once the diffusional contribution to selectivity (coming from the different ionic mobilities) is set aside, the experiments with OmpF mutants suggest that certain protein residues are responsible for the specific interaction of divalent cations with the protein. The binding of divalent cations to large channels can be of importance for the translocation of molecules with antimicrobial properties across bacterial porins [80]. In the case of OmpF channel, such a binding has been reported to favor antibiotic permeation [81, 82]. Other channels like lysenin have a binding site for divalent cations too [78], which allows using the lysenin channel as a biosensor for multivalent cations [83]. We have also shown that single channel measurements conductance over a wide range of salt concentrations make it possible to separate the intrinsic properties of the 2 : 1 electrolyte itself and other short range interactions of divalent cations with the protein.

## Figures and Tables

**Table 1:** Ion selectivity of OmpF (WT and mutants) in KCl and CaCl_2_.

OmpF channel	Δq*	RP (mV) 1/0.1 M KCl	RP (mV) 1/0.1 M CaCl_2_	Selectivity inversion
WT	0	−25.4 ± 0.8	22.1 ± 0.7	Yes
CC	+2	−23.8 ± 0.8	30.1 ± 1.1	Yes
RR	+4	31.9 ± 1.0	35.4 ± 1.7	No

*Δ*q*: effective charge compared to WT OmpF.
